# Medical Topography, Climate and Disease of Western New York

**Published:** 1838

**Authors:** F. H. Hamilton

**Affiliations:** Auburn, New York


### Art. II.—Endemic Fevers of New-York.

*Some account of the Medical Topography, Climate and Endemic Fevers, of the Western part of the State of New-York.* Extracted from a Discourse delivered before the Cayuga Medical Society, March 1837; By F. H. Hamilton, M. D.

The State of New-York, is divided geographically by several ranges of mountainous elevations which pass nearly east and west, through the southern tier of counties, and by the western branch of the Catskill, which intersects these at almost right angles, crossing the Mohawk at Rockton. The surface of the country north and west of these lines, has the aspect of its inclination chiefly north, most of its rivers emptying into Lake Ontario, and constitutes, according to our division, a portion of the middle territory. This section of the State is interspersed with numerous lakes and many extensive marshes. The principal lakes are, Chataque, Hemlock, Conesus, Honeoye, Canandaigua, Crooked, Seneca, Cayuga, Owasco, Skaneateles, Otisco, Onondaga, Cazenovia, Oneida, Otsego, Erie and Ontario; besides these, there are numerous other smaller basins of water. The principal marshes are the Tonewanto, Cayuga, Onondaga, Newton, Sodus, Catharines and Braddocks Bay: most of these lie along the outlets of small Lakes; some of them, as the Onondaga and Cayuga, cover several thousand acres. The former being about seven miles in length and three in its greatest breadth; the latter about eleven miles in length, and two and a half in breadth.

Notwithstanding the great amount of surface which is thus covered with water, we are in a great measure exempt from the miasmatic fevers as they appear in the more western states, or the interior of the middle territory. The surface of the country is more than usually undulating, considering its geological structure; the lakes though sluggish, are generally remarkably clear and deep; and the climate, though proverbially inconstant, is colder than in the same latitudes farther west, owing to its great northern and western exposure, and its chain of mountains on the south and east, which intercepts the warmer breezes of the southern and eastern latitudes. Eastern winds are of rare occurrence, and generally bring rain or snow. The southern winds are more frequent, and invariably damp and chilling for the first forty-eight hours, blowing during this time from the frosty summits of the Susquehanna and Alleghany mountains. When the air reaches us from the Gulph of Mexico and tropics, a rapid change takes place, the cold being succeeded by a genial and often sultry warmth. The prevailing winds are north and west, which are cool and invigorating, and are always hailed as the harbingers of a clear sky and healthy air. The Thermometer generally ranges in winter, from 10° below zero, to 50° above of Fahrenheit—in summer from 50° to 96°.

These are the most important circumstances which have always rendered the Lake country, or pleasant Valley, as the Indians termed it, remarkable for its salubrity. The Iroquois, the ancient lords and occupants of this soil, were a race distinguished above all other aborigines of the continent, no less for their prowess in war, their manly vigor and strength, than for their superior intelligence in council. The Tuscaroras only among them, were considered effeminate; but this tribe migrated from the south, and received from the land of their origin, an hereditary physical and mental inferiority. The early settlers, although they chose to locate along the valleys and in the vicinity of lakes, were hardy and robust: very few, comparatively, contracted the fevers peculiar to new countries, and they were seldom characterized by the same virulence, which at present distinguishes the fevers of Illinois and Missouri. The only remarkable exceptions have occurred in the years 1796, 1801, 1811, 1821–2; each successive epidemic having returned at somewhat regular intervals, varying from five to ten years. The fevers of 1811, were followed in 1812–13 and 14, by the pneumonia typhoides, a new and fatal epidemic, whose terrible ravages were almost confined to the nothern states. From this time until 1821, the endemic fevers seemed almost extinct, when they again returned with unparallelled severity and malignancy. During the last seventeen or eighteen years, their frequency and fatality have gradually abated, and they have now nearly disappeared from the more healthy localities, and are only to be found lurking in the most intensely miasmatic districts. And as the cyles of their return have nearly twice gone by, and the sources of their development are mostly extinguished, we may, with gratitude, hope that we shall no more be awed by their scourging visitations. The few rare cases which now and then occur, assume chiefly the type of the mild tertian, occasionally the quotidian, rarely terminating in the continued and typhus. They mostly prevail in the autumn, in the months of September and October; the cases which accasionally occur in the spring, we have many reasons to suppose are the consequence of a poison imbibed the preceding fall, having remained latent during the severity of the winter, which season is peculiarly unfavorable to its development. In the summer months, they generally assume the remittent and continued type, and are liable to terminate in a typhoid state.

In pursuance of our plan, we must here be permitted to reduce the hitherto general description, to a more minute analysis of the minor sections, and exhibit as far as practicable their local peculiarities, tracing from these, in the positive relation of cause and effect, the most prevalant type of their fevers.

The broad basin of the Mohawk, often inundated by the spring floods, from the highlands at its source, and extending many miles into the argillacious soil of the middle territory, is, at no time, scarcely less healthy, than the summits of the neighboring Catskill. A few cases of intermittent occur every year along its banks, and particularly at Utica, in the vicinity of which is the extensive Oneida jungle, the effluvia from which are often floated over it by the prevailing west winds, producing, since the intermediate forests have been removed, considerable autumnal intermittent fever, particularly in the west part of the city which lies low and most exposed; and even here during the last few years, it has again become very rare.

Syracuse, situated in the very midst of a salt marsh, is bounded on all sides except the north, by considerable hills; a low mucky soil extends in this direction to the shelving shores of the Onondaga Lake. By the mixture of its own fresh water with the numerous salt springs in its vicinity, it becomes the fertile source of miasmatic poisons, and has always been the very hot-bed of pestilence. The inhabitants are therefore more or less subject to intermitents and remittents, at all seasons of the year. The fevers often terminate rapidly in typhus, indicating a great concentration of the miasma. Many who become residents, acquire the same acclimation by a series of chills, as do the settlers in new countries. In 1821–2, the prevailing epidemic showed itself with unusual severity at Syracuse, and its neighboring village Salina. At Syracuse, many died suddenly, the disease assuming often the most distructive characters of Yellow fever, being accompanied with a deep yellow skin and vomiting of black blood, when fatal generally terminating in three or four days. At Salina, several deaths occurred at the same time, with singular phenomena, complicated with great determination to the head or bowels, and in the latter case followed by bloody dejections. The Cholera also, which seems to seek out the same localities with miasmatic fevers, made fearful havoc, both at Syracuse and Salina.

In the county of Cayuga, we occasionally meet with these fevers in the healthy yeomanry of the southern towns, but they prevail endemically only along the northern outlets of the Lakes; which uniting, form the slow and almost stagnant Seneca; which from its numerous, and partially drained bayous; its broad and half inundated marshes, sends forth an eternal miasmatic fog, stamping the character of all the diseases in the vicinage. At Montezuma, situated on the marsh of the same name, the declination of the river is scarcely one inch per mile; and its channel can hardly be distingnished from the marsh itself, as it winds its sluggish course through the exuberant jungle growths. Some attempts have been made to drain and till these marshes, more rich and fertile than the celebrated fields of Argolis, but they have hitherto failed, and it would require, we fancy, a more potent than even the famed son of Jupiter and Alemena, to accomplish such an enterprize. Salt springs also abound at Montezuma, which tend still further to counteract the miasm. In 1821, the congestive intermittents proved very fatal to the intemperate Irish, employed on this section of the works, sweeping them off in scores: at Jacks reefs, lower down on the same river, additional hands were required to pump the water from the Canal as it oozed thro' its loose banks, and such havoc was daily made that, for a time, the contractors were compelled to relinquish the work for want of laborers. Their fevers, are of course, of a low character, seldom assuming the high grades of synocha. They generally present a marked predominance of gastric and hepatic derangement, seldom terminating fatally in the first attack; but by repeated returns, the stomach and liver become essentially impaired, and the patient ultimately dieing with chronic dyspepsia or tuberculous liver.

At the Villages of Port Byron, Weedsport, &c. they constitute the prevailing diseases, and in a great measure prevent the rapid settlements, which many other local advantages might seem to encourage.

Auburn, situated on the Owasco outlet, sufficiently remote from the lake not to feel its malignant influence, the surrounding country mostly elevated and airy, has always enjoyed the most remarkable immunity from fevers. In December 1806 at Palmyra, and in many other places in general considered healthy, a fever of a typhoid character prevailed with great fatality, being accompanied with great prostration, coma, delirium, subsultus tendinum, hiccough, &c., those who died, seldom surviving the 3d or 4th day; but at Auburn the fevers were no more than usually malignant. The pneumonia typhoides, which in 1812, 13 and 14 swept over the country through its breadth and length, unstayed by human skill, scarce found a victim in our then new but populous village. Numbers were attacked, in and about the limits of the town, but in a form so much mitigated as to be easily controlled by the prompt application of the proper remedies. At east Cayuga, where a small garrison of United States troops were for a time quartered, amounting to about thirty, it showed itself with great severity and fatality. The prevailing epidemics of 1821 and 2, were equally unknown with us, nothing very unusual being presented during these years, in the general character of our fevers. And with the same perfect security did we stand and contemplate the terrible havoc of the eastern pestilence in our sister villages. For days its chariot of death rolled heavily along our borders; the tears of the disconsolate widow and bereaved orphan, marked its track. We made no *cordon sanitaire*, but the shield of God was about us; we felt not its iron grasp nor witnessed its fearful scenes. But a few years since, when our population numbered over 4000, during three months, the village burial ground was not opened nor a grave dug, nor the sound of the funeral bell heard in our streets: such is the remarkable salubrity of this favoured spot. At present, miasmatic fevers are almost unknown, such as do occur in the spring and autumn, are mostly in residences situated along the Owasco river, and are mild and easily subdued. Even those portions of the town will be rendered much more healthy by the completion of the splendid hydraulic works, undertaken and now nearly accomplished by its enterprising citizens. By which improvement, the river has been very much enlarged and deepened, and made to cover a considerable extent of low bottom, from which at dry seasons of the year, more or less miasmatic poison was extricated; and by which as perhaps a no less beneficial result, the numerous and crowded little huts along its course through the lower parts of the town, are in the way of being removed and replaced by the company, with larger and better constructed buildings, for various hydraulic purposes. The erection of a large and commodious stone market, which, as a specimen of architecture, is unsurpassed by any building of the kind we have ever seen, must also be regarded as a hygienic circumstance of vast importance, by leading to the removal to remote parts of the town, of the several lay stalls, formerly built over different parts of the river, the offals from which were thrown into the stream and floating against its banks, tainted the air with noxious effluvia. We are not ignorant, while making this statement, of the recent laborious researches of Parent Duchatelet, in and about the environs of Paris; in the course of which he visited "that most abominable sewer-like stream, the Rivere de Bierre near the Gobelins" upon which were situated nearly all the slaughter-houses of Paris, and especially that "incomparable stable of filth and nastiness" the enormous *chantierres d'quarrisage* of Montfaucon, where not only 12 or 13,000 horses, 12,000 dogs and 6,000 cats are killed yearly, but almost all the ordure of Paris is collected together." And yet he assures us of the remarkable healthfulness of the families who are employed, and who live in the vicinity. He tells us of the "*sante la plus florressante*" of the men, the "*sante brillante*" and the "*fecondite remarquables*" of the women, and the "*force et mine admirables*" of the infants, who are sometimes cradled "*dans c'interieur d'une carcase, comme dans un bureau?*" We have read the report of M. Duchatelet with interest and attention, but confess ourselves not at all convinced of the accuracy of his statements, or the correctness of his conclusions. A statement however of our grounds of dissent would transcend the proposed object of the present dissertation. We have dwelt the more particularly upon this point, because we believe that physicians and others who are made the conservators of the public health, are not sufficiently aware of the harm of those and similar establishments, and therfore permit their location in the populous parts of towns, to the great detriment of the comfort and healthfulness of the whole neighborhood.

At Seneca Falls, Geneva, Canandaigua, &c. endemic fevers are more common than at Auburn, yet scarcely less mild. In 1792, during one of the fall months, every person, male and female, in the village of Geneva was sick with fever except one, a young woman.

Rochester in its locality and diseases resembles Utica. The adjacent country is low and swampy, into which the western portion of the city partly extends. Intermittent fevers are however already becoming less common, and will still continue to decrease as the lands in the vicinity, yet new, become more drained by exposure and cultivation.

At Buffalo, they prevail chiefly in the lower parts of the city, where, from the rapidly increasing population, the streets have been extended into the very midst of the marshes before they were drained or hardly cleared. Many houses in the suburbs are erected on piles, and the green frog is permited to croak his dismal song unmolested in the fen beneath. The cholera made here a fearful tarry, and at no place were its victims more violently attacked.

The few cases of endemic fever which occur at Oswego are generally near the southern limits of the town, where the ground is cold and marshy, and seem not to depend upon their vicinity to the lake, for in the more populous parts of the village, or the harbor, its occurrence is comparatively rare; a fact of some importance as indicating the real estimate we are to place upon such collections of water in the production of such fevers.

North of Oswego, towards the St. Lawrence, the latitude is too high for the developement of miasma in a much concentrated form. The Black river in Jefferson co. which brings from the mucky soil, from which it has its origin, abundance of vegetable mould, and throws it at certain seasons upon its low bottoms, would doubtless prove a fruitful source of malaria, and of high grades of intermittent and remittent fevers in a more southern climate. But few cases occur, however, either at Watertown, or Sacket's Harbor which stands on the Bay at the mouth of the river.

Having now given a cursory glance at the principal localities in the state of New York, where endemic fevers more or less prevail, and described as far as our limits will permit, their peculiar geographical features, and the general character of their diseases, we retrace our steps to mark the therapeutic treatment applicable to these different sections. For the better attainment of which purpose, we propose to arrange their localities in three classes: beginning with those in which the malaria appears to be most abundant. In this class we place Syracuse, Salina, Weedsport, Port Byron, Montezuma and the lower parts of the city of Buffalo, with such other places not enumerated, as are similarly situated. Our description of symptoms and also of treatment, must necessarily be brief: indeed we prefer always the statement of a few striking diagnostics, and the selection of the most valuable and best attested remedies, to an endless assemblage of phenomena and a learned enumeration of obsolete and useless recipes.

In the districts just mentioned, the fevers, it will be seen, are characterised by a marked predominance of hepatic and gastro intestinal derangement. The attack is often sudden; and whether the fever be about to assume an intermittent or remittent form, it commences with a general lassitude slight nausea, constipation, head-ache, vertigo, and often an obtuse pain in the right hypochondriac region. These symptoms are succeeded more or less rapidly by a violent chill, which is soon followed by a burning fever, attended with nausea and sometimes vomiting of a green or yellow bile, throbbing pain in the head, bounding pulse, urgent thirst, restlessness, &c. If the attack be only a paroxysm of intermittent, the skin after a time becomes moist, and the febrile symptoms abate or almost entirely disappear. If, however, it pass into a remittent type, no perspiration occurs, the skin becomes more heated, the eyes suffused, the sleep disturbed, the fever continuing to augment and during the night being attended with slight delirium; towards morning a remission of all the symptoms takes place; in the afternoon a second paroxysm occurs, attended with more violent symptoms and is in the morning followed by another remission. After a continuance of these phenomena, during four or five days, the countenance acquires a more decided jaundice hue; the matter ejected changes from a yellow to a brown color; the tongue and teeth become loaded with a dark fur; the delirium increases, a clammy sweat appears on the surface, having a peculiar cadaveric fetor, subsultus tendinum and hiccough succeed, the patient sinks into coma, and death closes the scene.

However much Broussais and his followers may denounce the use of emetics and calomel as "unpathological unscientific and horrible" we believe them in such an assemblage of symptoms wholly indispensable. They are the Ajax and the Hercules in the hands of the skilful physician, and must be employed if he would conquer this formidable fever. The timely administration of an ipecac and ant. emetic, followed after a few hours by a brisk cathartic of calomel and jalap, will often at once disgorge the liver, evacuate the alimentary canal and restore to a healthy action the whole glandular system. Generally a single liberal bleeding may be premised, and occasionally, when the force of the pulse will permit, in the subsequent course; but we must constantly bear in mind in these cases, the tendency to prostration and a typhoid state, and must, therefore, endeavor neither to fall into the excessive purgation of Hamilton, nor the indiscriminate sanguineous depletion of Rush. After a thorough evacuation by emesis and catharsis, we should stay our medicines a little and observe their effect. The system of goading the stomach constantly and unremittingly, we deprecate as unscientific and irrational. A temporary suspension of hostilities should occasionally be allowed, that we may give the enemy an opportunity to evacuate the garrison quietly ; and the interval of truce may well enough be filled up with soothing and demucent articles, such as gum arabic water, rice water, &c., and external evaporants or irritants, according as special symptoms may indicate. If, after a proper interval, the disease appear still unsubdued, the attack may be repeated in such a form as shall be adapted to the character it now assumes.

If the disease be not arrested and the stage of prostration approach, no time can be lost; a few hours may place the patient beyond the reach of human skill. Quinine and opium are now the anchors of hope; when timely cast, they will stay him in the ebbing flood of life, when all others fail. From 1-2 to gr. j. of opium every six or eight hours; and quinine according to the indications of the pulse and susceptibility of the stomach. As adjuvants, we may employ Madeira wine or Brandy, carbonate of Ammonia, Fowler's solution, beef broth, or essence of beef. In the last of which we have perhaps the most concentrated and most convenient possible form of nutriment: consisting of pure serum, and ozmazome the essential pabulum of all animal foods. It is prepared as follows: A piece of the surloin of a fat beef, two or three days killed, cut into small pieces about the size of walnuts, and put into a tightly corked junk bottle; place the bottle in a kettle of cold water over a fire, and let it boil half an hour. From a pound of meat thus boiled, half a teacupfull of rich soup may be obtained; strain it through a coarse muslin cloth, add a fraction of red pepper and salt, also a little mace. *Medium dose:* A desert-spoonfull every half hour.

In the second class, we place Utica, Seneca Falls, Rochester, Lockport, Oswego, &c. The fevers in these localities differ from the same in the places before mentioned, by assuming a less bilious and more decidedly inflammatory character. The onset is generally not less rapid than in more miasmatic districts, but is seldom preceded by that *extreme* sense of langor and debility, consequent upon a gradual derangement of the hepatic function. Early local determinations are the most characteristic features. Inflammations of the brain, lungs, pleura, liver, bowels or some other important organ, being almost invariably present. The functions of the liver are often no more impaired than the functions of every other organ. Now and then the fever is of a mixed character, exhibiting either through its whole course, or only occasionally, especially after a long continuance of the febrile symptoms, a strong tendency to disease of the liver, the stools varying from almost black, to dark green or yellow; the countenance becoming icterode, and the tongue acquiring a brown fuliginous fur. The most common termination of the fever however, if not speedily arrested, is in local congestion and effusion. The approach of this stage is characterized by a train of symptoms, varying according as one or another organ is the seat of the effusion. When the brain is about to become the seat of this lesion, it is indicated by diminished force of pulse, torpor of bowels, dull, obtuse pain in top or back of head, accompanied with a sense of weight or distension; a tendency to stupor, slight mental aberrations on being suddenly aroused from sleep, which rapidly passes into almost total insensibility, the pupil becoming dilated, and the features contracted and cadaveric. When the lungs are the organs which have chiefly suffered, we are warned of serous engorgements of the air cells and parenchyma by a sudden subsidence of the severe pectoral signs of inflammation, short and labored respiration, an abundant, liquid, transparent expectoration, loose mucous rattle and other physical signs recognized by the stethescope. When this condition after a short and severe course suddenly supervenes, the indication is fatal; medicines do neither arrest, nor even stay its uniform course. Our only remaining duty is to apprise the patient and his friends of the inevitable result, and commend him to God, the great physician of souls. When however effusions occur as sequelæ to a more protracted course, they may be presumed to be unaccompanied by those apoplectic engorgements and sanguine extravasations which are the usual concomitants of the acute form, and to be the effect alone of an excessive debility of the capillary system, a degree of lesion which is often susceptible of reparation.

In the acute stage, the leading indications of treatment are early, liberal, and frequent bleeding, followed by a single cathartic of calomel and jalap; and occasionally under peculiar circumstances and after sufficient depletion, a gentle emetic. Blisters are scarcely admissible earlier than emetics, and require the same precautions; if applied too early, they seldom fail to aggravate the general fever and local inflammation. Topical or capillary bleeding, so much cried up by the French, and lauded by many of our own writers, cannot be trusted. In large cities where the fevers seldom assume the most acute grades of local inflammation, such measures may prove valuable as adjuvants; and scarification and leeching may continue to terrify and torture their pale and exsanguineous patients; but their general introduction into these districts, will only serve to deceive the physician and pacify the patient, until fatal disorganization ensues and the fate of the patient is sealed.

In the chronic form, when the occurrence of effusion is suspected, the *methode expectante* rather than the *methode empirique* should be adopted, the transition from one stage to another being often so gradual and imperceptible as to leave the most experienced in doubt where depletion should cease and stimulation commence. But the crisis being fully ascertained moderate excitants are demanded. Calomel and blisters here display their greatest power, the former working by virtue of its influence over the secernent and absorbent systems, the latter acting both as a revellent and general stimulant, relieving the oppressed organ, and arousing at the same time the nervous and arterial energies.

The third class includes Auburn, Geneva, Palmyra, Canandaigua, &c. Here at the present day, we are almost out of the reach of miasmata, except what the lakes and a few stagnant pools in our vicinity may engender, and this is so little as scarcely to distinguish our fevers from those which annually more or less prevail in the New England States. They cannot, therefore, be rightly considered as belonging to the endemic fevers of the middle territory, since they retain none of the essentially diagnostic marks. The chief of which is always *more or less hepatic derangement.*

We shall, therefore, only say by way of description, that here are found those fevers with which Broussais was more particularly familiar and upon which he based his doctrines of gastro enteritis as the universal source of fever. Let any one acquainted with the fevers of these places read, the general description of fevers given by Boisseau, the faithful, but not servile follower of Broussais, and he will soon become convinced that such is the fact. We do not think that in a practice of several years in this district, we have seen a single case which did not present marked symptoms in the very onset of gastro intestinal irritation. In forming which conclusion we disclaim entirely the influence of any prejudice in favor of Broussaism. Accordingly we have always adopted, to a greater or less extent the physiological practice as its advocates par excellence have termed it; and have never yet had reason to be dissatisfied with its results; (having never yet lost a patient with fever, nor witnessed a termination in typhus.) Calomel, the *sine qua non* with many, we have considered the *fons et origo mali*, and have used it with a sparing hand; and believe it the fruitful source of mucous disorganization and typhoid prostration, not to speak of the numerous other and lasting evils which it entails. We cannot surely be too severe in reprobating the horrible practice of universal salivation in our fevers. It is now every where the prevailing axiom *proritatio salivae febres sanabant*, hence this is ever the first and grand indication. Strange infatuation! so we believe it. The cause is mistaken for the effect and the effect for the cause. Does the fever abate because the calomel salivates? Nay, the calomel salivates because the fever abates. Every practitioner of twenty years experience in this country, has seen more cases than one when ptyalism would cease upon the approach of the hot stage, and regularly return on the approach of the sweating stage, and this for several days in succession; and yet have they been totally blind to the only legitimate conclusion, and have persisted in their irrational course until the whole system was saturated, and the whole fabric of the constitution undermined.

In the early settlement of these places endemic fevers were not uncommon, and calomel seemed often essential to success. Our fathers in the profession whose grey hairs have witnessed, in sad emblem, the sear and fallen leaves of many an autumn adored its power; it was to them "the mercury," the messenger of Jove, who alone could meet the destroyer, or unstring his bow and arrest his poisoned arrows. The homage, then, they continue to pay to him is natural and inevitable. Though we venerate age and its experience, we should not imbibe its prejudices and errors. We doubt not that mercury was to our fathers as a messenger from Heaven; but the grand cause of his embassy to us has now almost ceased; let our diplomacy therefore be more measured and circumspect, lest by our indiscreet intercourse we add to the many shattered and decrepid relics, which while they live, will remain as lasting monuments of the effects of mercurial ulceration—the curse and obloquy of our science: and through succeeding generations, long as their memory is retained by written history or fire-side tradition, must cause ignorance and empiricism to triumph over medical learning and genuine philanthropy.

Auburn, New York.

